# A randomized intervention involving family to improve communication in breast cancer care

**DOI:** 10.1038/s41523-021-00217-9

**Published:** 2021-02-12

**Authors:** Jennifer L. Wolff, Jennifer Aufill, Diane Echavarria, Amanda L. Blackford, Roisin M. Connolly, John H. Fetting, Danijela Jelovac, Katie Papathakis, Carol Riley, Vered Stearns, Nelli Zafman, Elissa Thorner, Howard P. Levy, Amy Guo, Sydney M. Dy, Antonio C. Wolff

**Affiliations:** 1 Roger C. Lipitz Center for Integrated Health Care, Johns Hopkins Bloomberg School of Public Health, Baltimore, USA; 2 The Johns Hopkins University School of Medicine, Baltimore, MD USA; 3 The Johns Hopkins Sidney Kimmel Comprehensive Cancer Center, Baltimore, MD USA

**Keywords:** Breast cancer, Health policy

## Abstract

We examined the effects of a communication intervention to engage family care partners on patient portal (MyChart) use, illness understanding, satisfaction with cancer care, and symptoms of anxiety in a single-blind randomized trial of patients in treatment for breast cancer. Patient-family dyads were recruited and randomly assigned a self-administered checklist to clarify the care partner role, establish a shared visit agenda, and facilitate MyChart access (*n* = 63) or usual care (*n* = 55). Interviews administered at baseline, 3, 9 (primary endpoint), and 12 months assessed anxiety (GAD-2), mean FAMCARE satisfaction, and complete illness understanding (4 of 4 items correct). Time-stamped electronic interactions measured MyChart use. By 9 months, more intervention than control care partners registered for MyChart (77.8 % vs 1.8%; *p* < 0.001) and logged into the patient’s account (61.2% vs 0% of those registered; *p* < 0.001), but few sent messages to clinicians (6.1% vs 0%; *p* = 0.247). More intervention than control patients viewed clinical notes (60.3% vs 32.7%; *p* = 0.003). No pre-post group differences in patient or care partner symptoms of anxiety, satisfaction, or complete illness understanding were found. Intervention patients whose care partners logged into MyChart were more likely to have complete illness understanding at 9 months (changed 70.0% to 80.0% vs 69.7% to 54.6%; *p* = 0.03); symptoms of anxiety were numerically lower (16.7% to 6.7% vs 15.2% to 15.2%; *p* = 0.24) and satisfaction numerically higher (15.8–16.2 vs 18.0–17.4; *p* = 0.25). A brief, scalable communication intervention led to greater care partner MyChart use and increased illness understanding among patients with more engaged care partners (NCT03283553).

## Introduction

Those living with serious illness commonly value, desire, and rely on family^1,2^. However, the family is typically overlooked in cancer care^3^. Few interventions have explicitly assessed and supported patients’ preferences for engaging family in communication at the point of care^4,5^. Many electronic health record vendors and care delivery organizations allow patients to share access to their patient portal account in a registration process through which a family “care partner” receives their own identity credentials (login/password)^6^. Shared (proxy) access to the patient portal may be particularly helpful in the context of cancer due to heavy reliance on family and the intense longitudinal demands of treatment^3^. However, awareness and uptake of shared access to the patient portal is low^7,8^. The implications of an engaging family in electronic cancer care interactions is not well understood with respect to the use of the patient portal, the ability to successfully manage the demands of cancer care, or quality of life.

We developed a communication intervention for patients who attend oncology visits with family or other unpaid care partners. “Sharing in Care” sets forth a structured process to establish a shared visit agenda and clarify expectations about the role of the family during in-person and electronic interactions with the care team. Our hypothesis was that appropriate engagement of care partners in electronic interactions through registration and use of shared access to the patient portal would reduce symptoms of anxiety while improving illness understanding and increasing satisfaction with cancer care. To test this hypothesis, we conducted a single-blind randomized trial of patients in active treatment for breast cancer and their care partners to examine between-group differences in study outcomes at 9 months. In our baseline analysis, we reported that Sharing in Care was found by patients and care partners to be easy to complete, useful, recommended for use by others, and led to greater care partner registration for the patient portal at 6 weeks^9^. This report of the final results describes effects on patient and care partner use of the patient portal and on measures of symptoms of anxiety, satisfaction with cancer care, and illness understanding at 9 months.

## Results

### Study flow

**Fig. 1:**
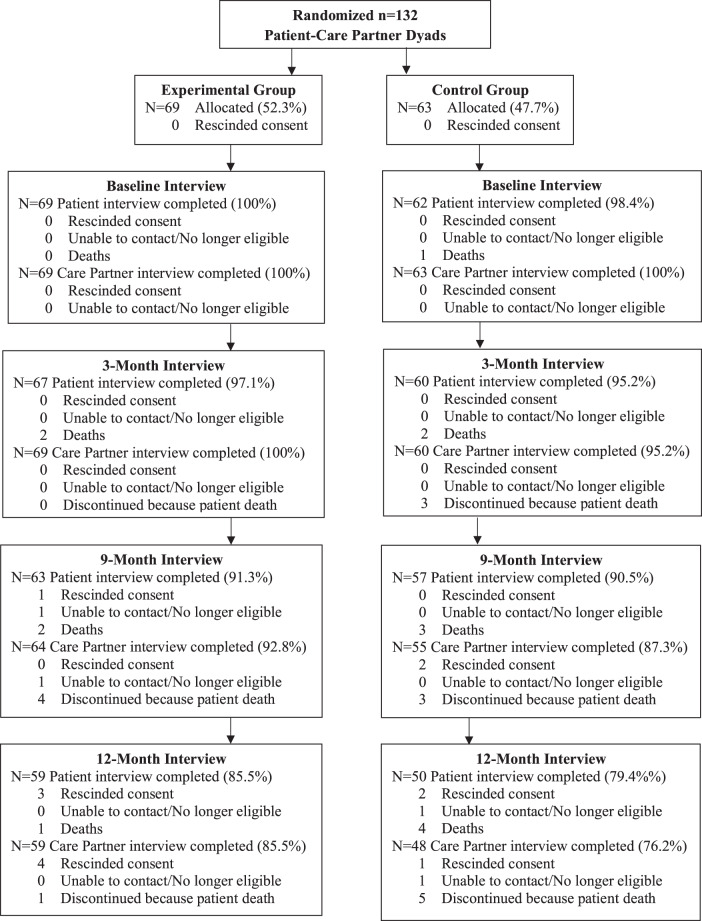
Participant flow. Sharing in care CONSORT diagram.

Figure 1 displays the flow of study participants. Recruitment letters were mailed to 361 patients of participating clinicians. Twenty (5.5%) returned a mailed card indicating that they were not eligible (*n* = 11; 3.0%) or declining participation (*n* = 9; 2.5%); 21 (5.8%) could not be reached. Screening calls were fielded to 320 patients of whom 139 (43.4%) were not eligible and 49 (15.3%) refused participation: 132 (41.3%) patient-companion dyads were eligible and agreed to participate. Enrolled patients were younger than those who did not participate (53.9 versus 56.8 years; *p* = 0.045) with no other differences observed.

Enrolled dyads were randomized to the control (*n* = 63) or intervention group (*n* = 69). At 9 months (primary endpoint), 57 (90.5%) control patients plus 55 (87.3%) care partners and 63 (91.3%) intervention patients plus 64 (92.8%) care partners completed follow-up interviews. Twelve patients (six intervention and six control) and 13 care partners (five intervention and eight control) discontinued participation by 9 months, primarily due to the death of the patient (*n* = 10; 7.5%). One (0.7%) intervention patient and 2 (1.5%) control care partners rescinded consent and 1 (0.7%) intervention dyad was lost to follow-up. Fifty-nine intervention dyads and 48 control dyads reconsented and completed interviews at 12 months. No harms were observed or reported.

### Study sample characteristics

There were 118 dyads with complete assessments at 9-months (Table 1). Enrolled patients had a mean age of 53.5 years. Most were women (99.2%) with a high school education or more (87.3%). About half (56.8%) were diagnosed with early stage disease. Two thirds of care partners were men (65.3%) who were spouses or partners of patients (60.2%). Care partners were also adult children (14.4%), or friends or other relatives (25.4%). Participant characteristics were similar by group, except intervention patients were more likely than control to have beyond high school education (93.7% vs 80.0%; *p* = 0.03) and intervention care partners were less likely than control to be male (57.1% vs 74.6%; *p* = 0.05). Daily internet use by patients and care partners was comparable.

**Table 1:** Study participant characteristics by group assignment.

	Intervention (*n* = 63)	Control (*n* = 55)	Total (*n* = 118)	*P* value^b^
Patient characteristics				
Mean age (SD), y	54.7 (13.3)	52.2 (13.9)	53.5 (13.6)	0.318
Female gender, *n* (%)	62 (98.4)	55 (100.0)	117 (99.2)	0.348
Nonwhite race or hispanic, *n* (%)	15 (23.8)	17 (30.9)	32 (27.2)	0.387
Beyond high school education, *n* (%)	59 (93.7)	44 (80.0)	103 (87.3)	0.026
Suspected of low health literacy, *n* (%)	7 (11.1)	4 (7.3)	11 (9.3)	0.474
Married, *n* (%)	41 (65.1)	39 (70.9)	80 (67.8)	0.499
Early stage disease, *n* (%)	34 (54.0)	33 (60.0)	67 (56.8)	0.389
Node negative (N0)	20 (58.8)	16 (48.5)	36 (53.7)	0.396
Phenotype				0.245
ER positive/HER2 negative	17 (50.0)	12 (36.4)	29 (43.3)	
HER2 positive	13 (38.2)	12 (36.4)	25 (37.3)	
Triple negative	4 (11.8)	9 (27.3)	13 (19.4)	
FACT-B Score, (SD)^a^	115.0 (22.6)	116.3 (21.5)	115.6 (22.0)	0.752
Daily internet usage, *n* (%)	53 (84.1)	46 (83.1)	99 (83.9)	0.942
Care partner characteristics				
Mean age (SD), y	53.7 (13.7)	53.6 (14.0)	53.7 (13.8)	0.959
Male gender, *n* (%)	36 (57.1)	41 (74.6)	77 (65.3)	0.048
Beyond high school education, *n* (%)	57 (90.5)	46 (83.6)	103 (87.3)	0.266
Suspected of low health literacy, *n* (%)	14 (22.2)	15 (27.3)	29 (24.6)	0.525
Relationship to patient, *n* (%)				
-Spouse/Partner	35 (55.6)	36 (65.5)	71 (60.2)	0.477
-Adult Child	11 (17.5)	6 (10.9)	17 (14.4)	
-Other (e.g. parent, sibling, friend)	17 (27.0)	13 (23.6)	30 (25.4)	
Worked in the past week, *n* (%)	35 (55.6)	37 (67.3)	72 (61.0)	0.193
Daily internet usage, *n* (%)	55 (87.3)	46 (83.6)	101 (85.6)	0.572

^a^Quality of life measured using FACT-B: higher values = higher QOL (total score of 164).

^b^*P*-value for *χ*^2^ test of categorical characteristics, t-test for discrete continuous characteristics.

### Patient portal use

Patient and care partner registration for MyChart was similar at baseline by group (Table 2). At 9 months, intervention (versus control) care partners were more likely to be registered for the patient’s MyChart account (77.8% vs. 1.8%; *p* < 0.001). Of 48 intervention care partners who registered for MyChart during the study, most (*n* = 42; 89.4%) registered on the day of enrollment (range 0–14 days). Intervention care partners who were registered for the patient’s MyChart account were more likely to have logged into the patient’s account at 9 months than care partners in the control group (77.8% vs. 0%; *p* < 0.001). Intervention care partners who were registered for the patient’s MyChart account viewed patient messages (44.9%), test results (42.9%), and clinical visit notes (38.8%), but few (6.1%) engaged in messaging with clinicians using their own identity credentials. Among patients who were registered for MyChart, those in the intervention (versus control) group were more likely to view provider clinical notes (63.3% vs. 36.0%; *p* = 0.003). No other group differences in patient use of MyChart were observed.

**Table 2:** Effects on patient and care partner registration and use of the patient portal.

	Patient			Care partner		
	Intervention *n* = 63	Control *n* = 55	*P* value^a^	Intervention *n* = 63	Control *n* = 55	*P* value^a^
Registered for patient portal^b^						
Baseline	58 (92.1)	48 (87.3)	0.545	1 (1.6)	1 (1.8)	>0.999
9 Months	60 (95.2)	50 (90.9)	0.470	49 (77.8)	1 (1.8)	<0.001
Use of patient portal at 9 Months, n (%)^3^						
-Logged in to patient portal	59 (98.3)	50 (100)	0.732	30 (61.2)	0 (0.0)	<0.001
-Viewed clinical notes in patient portal	38 (63.3)	18 (36.0)	0.003	19 (38.8)	0 (0.0)	<0.001
-Viewed messages in patient portal	58 (96.7)	50 (100)	1.000	22 (44.9)	0 (0.0)	<0.001
-Viewed test results in patient portal	58 (96.7)	48 (96.0)	0.544	21 (42.9)	0 (0.0)	<0.001
-Exchanged direct message in patient portal	52 (86.7)	38 (76.0)	0.128	3 (6.1)	0 (0.0)	0.247

^a^*P*-value uses Fisher’s exact test to assess between-group differences in proportion of participants who used a patient portal feature at least once during 9-month follow-up period.

^b^Baseline refers to registration for the patient portal at the time of study enrollment; 9 months refers to patient portal activities at 40-weeks post-enrollment.

^c^Use of patient portal at 9 months is limited to participants who were registered for the patient portal.

### Patient and care partner-reported outcomes

**Fig. 2:**
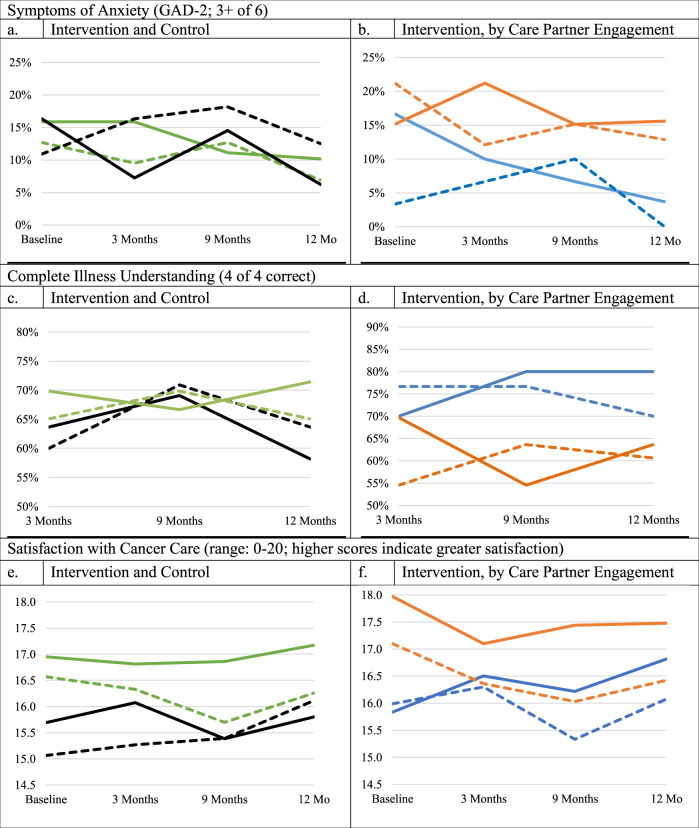
Between group differences in patient and care partner-reported outcomes. **a**, **c**, **e** Solid black line—Control Patient Dashed black line—Control Care Partner. Solid green line—Intervention Patient. Dashed green line—Intervention Care Partner. **b**, **d**, **f** Solid blue line—Patient with Engaged Care Partner. Dashed blue line—Engaged Care Partner. Solid orange line—Patient without Engaged Care Partner. Dashed orange line—Nonengaged Care Partner. Symptoms of anxiety refer to a cutpoint of 3+ on the GAD-2. Illness understanding ranges from 0 to 4 with higher scores indicating greater illness understanding: we compare participants with ratings of “4”, reflecting “complete illness understanding” with all others. Satisfaction with cancer care is measured using the FAMCARE (range: 0–20) with higher scores reflecting greater satisfaction. Intervention versus control group outcomes: *N* = 118 (*n* = 63 intervention, *n* = 55 control). Intervention group outcomes, stratified by whether the care partner accessed MyChart: *N* = 63 (*n* = 30 with an engaged care partner, *n* = 33 without engaged care partner).

No between-group differences in patient or care partner-reported symptoms of anxiety, illness understanding, or satisfaction with cancer care were found over the observation period (Table 3; Fig. 2). The proportion of patients characterized as having symptoms of anxiety decreased between baseline and 9 months from 16.4 to 14.6% in the control group and from 15.9 to 11.1% in the intervention group (interaction *p* = 0.62). The percentage of patients with complete illness understanding changed between 3 and 9 months from 69.8 to 66.7% in the intervention group and from 63.6 to 69.1% in the control group (*p* = 0.26). Patient satisfaction with cancer care was relatively stable for both the intervention (mean (SD): 17.0 (3.5) and 16.9 (3.9)) and control group (15.7 (4.5) and 15.4 (5.4)) between baseline and 9 months (*p* = 0.56).

**Table 3:** Comparison of outcomes at 9 months by group assignment and intervention care partners’ Use of MyChart.

	Patient				Care partner			
	BL	9 M	Estimate^b^ (95% CI)	P-Value^a^	BL	9 M	Estimate^b^ (95% CI)	P-Value^a^
**Intervention versus the control group**								
Symptoms of anxiety present (%)^c^								
Intervention (*n* = 63)	15.9	11.1	0.66 (0.27, 1.62)	0.619	12.7	12.7	1.00 (0.37, 2.73)	0.405
Control (*n* = 55)	16.4	14.6	0.87 (0.47, 1.61)		10.9	18.2	1.86 (0.64, 5.37)	
Complete illness understanding (%)^d^								
Intervention (*n* = 63)	69.8	66.7	0.83 (0.43, 1.58)	0.264	65.1	69.8	1.31 (0.60, 2.85)	0.532
Control (*n* = 55)	63.6	69.1	1.39 (0.74, 1.62)		60.0	70.9	1.87 (0.84, 4.16)	
Mean satisfaction with cancer care (SD)								
Intervention (*n* = 63)	17.0 (3.5)	16.9 (3.9)	−0.10 (−0.70, 0.51)	0.555	16.6 (3.8)	15.7 (4.4)	−0.87 (−1.62, −0.13)	0.108
Control (*n* = 55)	15.7 (4.5)	15.4 (5.4)	−0.31 (−1.35, 0.73)		15.1 (4.9)	15.4 (5.5)	0.33 (−0.67, 1.33)	
**Active intervention versus nonactive intervention**								
Symptoms of anxiety present (%)^c^								
Active intervention (*n* = 30)	16.7	6.7	0.35 (0.11, 1.11)	0.244	3.33	10.0	3.44 (0.62, 19.0)	0.131
Non active intervention (*n* = 33)	15.2	15.2	1.00 (0.27, 3.76)		21.2	15.2	0.65 (0.17, 2.47)	
Complete illness understanding (%)^d^								
Active intervention (*n* = 30)	70.0	80.0	1.89 (0.62, 5.71)	0.026	76.7	76.7	1.00 (0.28, 3.62)	0.578
Non active intervention (*n* = 33)	69.7	54.6	0.44 (0.23, 0.83)		54.6	63.6	1.58 (0.60, 4.17)	
Satisfaction with cancer care—mean (SD)								
Active intervention (*n* = 30)	15.8 (4.1)	16.2 (4.3)	0.39 (−0.58, 1.35)	0.250	16.0 (4.5)	15.3 (4.4)	−0.65 (−1.89, 0.59)	0.574
Non active intervention (*n* = 33)	18.0 (2.6)	17.4 (3.4)	−0.53 (−1.25, 0.19)		17.1 (3.1)	16.0 (4.4)	−1.07 (−1.93, −0.21)	

^a^*P*-value for differential changes between time point (9 months compared to baseline) and group assignment, adjusted for patient education, care partner gender, and patient disease stage for intervention compared to control, and adjusted for patient disease stage and care partner employment status for active intervention versus non-active intervention.

^b^Odds ratios (95% CI) for the presence of anxiety or complete illness understanding at 9 months compared to baseline and mean differences (95% CI) in satisfaction with cancer care at 9 months compared to baseline.

^c^Symptoms of anxiety refer to a cutpoint of 3+ on the GAD-2.

^d^Complete illness understanding contrasts scores of 4 (4 out of 4 questions answered correctly) versus all others at 3 and 9 months.

Between baseline and 9 months, symptoms of anxiety remained stable at 12.7% of intervention care partners and increased from 10.9 to 18.2% of control care partners (*p* = 0.41). The percentage of care partners with complete illness understanding increased from 65.1% to 69.8% of intervention care partners and increased from 60.0 to 70.9% among control care partners (*p* = 0.53). Care partner satisfaction with cancer care was numerically lower in the intervention group (mean (SD): 16.6 (3.8) and 15.7 (4.4)) and higher in the control group (15.1 (4.9) and 15.4 (5.5)); between-group differences were not statistically significant (*p* = 0.11).

### Intervention subanalysis by care partner engagement

Half of the care partners in the intervention group logged into MyChart during the observation period (*n* = 30 of 63; 47.6%). Care partners who logged into MyChart were more likely to assist patients with early stage disease (70.0% vs 39.4%; *p* = 0.02) and to have worked in the last week (70.0% vs 42.4%; *p* = 0.03) than care partners who did not. The percentage of intervention patients characterized as having symptoms of anxiety was lower at 9 months (16.7–6.7%; OR: 0.35, 95% CI: 0.11, 1.11) in dyads whose care partners logged into MyChart and remained stable at 15.2% at baseline and 9 months among patients whose care partners did not (*p* = 0.24). Complete illness understanding increased over the observation period from 70.0 to 80.0% (OR = 1.89; 95% CI: 0.62, 5.71) among patients whose care partners logged into MyChart and decreased from 69.7% to 54.6% (OR = 0.44; 95% CI: 0.23, 0.83) among patients whose care partners did not; this group difference was statistically significant (*p* = 0.03). Satisfaction with cancer care numerically increased (0.39; 95% CI: −0.58, 1.35) among patients whose care partners logged into MyChart and decreased (−0.35; 95% CI: −1.25, 0.19) among patients whose care partners did not log into MyChart (interaction *p* = 0.25). Care partner satisfaction with cancer care numerically decreased among those who logged into MyChart (−0.65; 95% CI: −1.89, 0.59) and those who did not (−1.07; 95% CI: −1.93, −0.21; *p* = 0.57). Observed subgroup differences in outcomes persisted through 12 months (Fig. 2).

## Discussion

A communication intervention to clarify the role of family care partners in face-to-face and electronic interactions led to meaningful increases in care partners’ access and use of the patient portal and twice as many patients viewing their oncology visit notes. No between-group differences were identified in patient or care partner symptoms of anxiety, illness understanding, or satisfaction with cancer care by 9 months. However, intervention patients whose care partners accessed MyChart using their own identity credentials during the observation period were more likely to have complete illness understanding than patients whose care partners were less actively engaged during the observation period. Our finding that a brief communication intervention at the point of care led to increased illness understanding among patients with more actively engaged care partners is of clinical importance for two key reasons.

First, longitudinal communication is an essential element of high-quality cancer care^10–12^. Timely, comprehensive, and accurate information about prognosis and treatment is valued by patients^13^ and families^14–16^, and contributes to illness understanding, shared decision-making, and delivery of care that is consistent with patient wishes^17–19^. Prior studies establish that those living with cancer rely on family throughout the illness trajectory, but that family is often poorly prepared for the demands of cancer care and caregiving^1,3^. Interventions for cancer caregivers have most often examined psychosocial support to address caregiver burden^4,5,20^. Developing approaches to support the family in cancer care has been an emerging area of interest but, with rare exception^21^, studies to date have most often addressed a specific visit or decision and been conducted outside^22–25^ rather than embedded in care delivery, as tested in our study.

Second, patient knowledge is foundational to a range of important outcomes such as informed decision-making, adherence, and receipt of burdensome care^17,26^. Interventions to strengthen patient capacity to manage their care typically require additional health professionals and staff, technologies, or changes to clinical workflows^27–29^. In contrast, the intervention we tested targets the person-family dyad directly by setting forth an easy-to-implement structured process to clarify roles that respect patient preferences and family contributions in co-managing care. Our approach employed existing health information technology functionality, addressed the identified need for strategies to more effectively engage cancer caregivers, and relied on strategies that can be easily scaled up for dissemination in real-world practice settings^5,30,31^.

In light of the accelerating spread of health information technology and increasing complexity of therapeutic regimens, identifying strategies that meaningfully engage families in cancer care through health information technology will increase in importance. Our study also reinforces evidence that merely deploying a technology does not assure therapeutic uptake or meaningful engagement. For instance, Southwest Oncology Group (SWOG) investigators recently reported randomized evidence showing that bi-weekly text message reminders alone did not improve adherence to adjuvant aromatase inhibitor therapy in breast cancer^32^. Designing care delivery interventions that involve user-centered co-design is critically important ensuring they resonate with and are meaningful to the intended audience.

Benefits of the patient portal operate through mechanisms of convenience, continuity, activation, and understanding^33^. Our findings suggest that for some, these pathways will be amplified when a care partner is also engaged. Overall, it is possible that the observed increase in patient illness understanding and shift toward reduced symptoms of anxiety could be partly due to clarifying role expectations and providing care partners with access to information, thereby setting the stage for more productive, honest discussion, information exchange, and support. Such a hypothesis is consistent with theory^34,35^ and evidence of clinical benefit from the purposeful engagement of care partners through technology^36–39^. While this conclusion would be supported by our finding that observed effects were more highly concentrated in the subset of patients with an actively engaged care partner, we did not test this specific hypothesis in our current study.

Our study provides insight as to how the uptake of shared access may affect care partners and clinicians. The convenience of direct access to patients’ health information may be especially important for those balancing multiple responsibilities, as suggested by higher rates of patient portal use found among working care partners. Busy clinicians concerned about the additional time demands of engaging with cancer caregivers should be reassured that, although registered care partners commonly accessed the patient portal to view patient health information, few engaged in direct messaging. In fact, shared access may benefit clinicians as it allows them to know with whom they are messaging (a patient or their care partner), and potentially reduce time demands due to care partners being able to directly access patient health information^40^.

Strengths of this study include its design, well-characterized sample, low attrition for reasons other than mortality, reliance on objective measures related to patient portal access and use, and collection of both patient- and care partner-reported information. This study is also subject to limitations. While we intentionally enrolled patients with early and advanced breast cancer, we did not design the study to differentiate intervention effects by disease stage. Also, while the study was conducted in two separate clinics (urban and suburban) within a single academic institution with a spectrum of patients that is representative of community practices, additional research will be needed to confirm the generalizability of our findings in mainstream oncology care. Finally, the widespread use of MyChart by patients in this study was higher than previous reports of modest registration^41^, which may be due to participants’ high levels of educational attainment. Higher levels of educational attainment among intervention patients may have partly influenced our findings.

In summary, we found a communication intervention to engage care partners at the point of care led to greater access and use of the patient portal among care partners, higher viewing of clinician visit notes among patients, and greater illness understanding among patients with more actively engaged care partners. The important role assumed by the family in navigating health system demands, participating in decisions, and ensuring treatment adherence is well established^20,42^. Our study demonstrates the feasibility and benefit of moving care delivery toward a person- and family centered principles^43^ that clarify and support patient preferences, legitimize care partner contributions, and afford appropriate information access to the range of family and other care partners who are so integral to enact high-quality cancer care.

## Methods

### Overall design

A CONSORT checklist is available as a supplementary file (See Supplementary Fig. 1). We conducted a two-arm, single-blind randomized trial of a communication intervention at two oncology clinics (one hospital-based and one community-based) within one academic health system that has a well-established integrated electronic medical record, Epic (Verona, WI) and MyChart patient portal (MyChart® is a registered trademark of Epic Systems Corporation). Medical oncologists and nurse practitioners were recruited between June and September 2017. Patient-family dyads enrollment began in August 2017 and completed in November 2018 and followed through November 2019. The patient-family dyad was the unit of analysis and randomization. All study procedures, consents, and surveys were reviewed by the scientific review committee of the Johns Hopkins Kimmel Cancer Center and subsequently approved by the Johns Hopkins University School of Medicine institutional review board on 05/19/2017 (IRB00129995). The study was first submitted to clinicaltrials.gov on 08/28/2017 and first posted on 09/14/2017 (NCT03283553). Recruitment procedures were previously described^9^.

### Study procedures

Oncology clinicians at participating clinics provided informed consent indicating their permission for the study team to contact their patients. Patients of participating clinicians who were in active treatment for early stage or advanced breast cancer were mailed letters describing the study three weeks before their next scheduled visit. Patients who did not “opt out” by mail were contacted by research staff to discuss study procedures and administer a screening interview. Patients undergoing active breast cancer therapy were eligible if they reported regularly attending appointments with a family or unpaid care partner who also agreed to participate. The goal was to enroll patients with early stage and advanced stage in equal numbers, approximately. Eligible patient-care partner dyads who expressed interest met a member of the research team at the clinic 30 min before the patient’s visit. Each patient and care partner dyad provided informed consent and was randomized using stratified, blocked randomization by clinician. Dyads assigned to the intervention were asked to complete the checklist immediately before the visit without instruction from research staff (See Supplementary Fig. 2). Dyads assigned to the control group received the usual care. Trained research staff fielded a standardized telephone survey to patients and care partners one week post-enrollment and at 3, 9, and 12 months.

### Intervention

A paper version of our patient-family agenda setting checklist was provided to patient and care partner participants for them to complete together at the point of care, immediately in advance of a regularly scheduled medical oncology visit. The checklist sets forth a structured process to establish a shared visit agenda and clarify expectations about the role of the family during in-person and electronic interactions with the oncology team, as previously described^9^. Upon completion of the checklist, front desk staff were instructed to implement participant’s stated patient portal registration preferences. After the visit, intervention patients and care partners were provided paper handouts with instructions on how to access MyChart and clinical visit notes and offered facilitated registration by research staff in the clinic^44^. Usual care refers to the availability of MyChart registration that may be self-initiated by patients and care partners under standard clinic protocol.

### Measurement

*Patient portal use* for both patients and care partners was assessed from the date and time-stamped interactions reflecting the frequency, timing, and type of MyChart interactions. Registration for MyChart was assessed at baseline and nine-months post-enrollment. Use of the patient portal refers to MyChart interactions between enrollment and up to 40-week post-enrollment, corresponding with the timing of our primary endpoint.

*Illness understanding* was measured by four questions regarding knowledge that is considered to be essential to making informed treatment decisions in serious illness, including (1) understanding of illness, (2) knowledge of disease status, (3) awareness of disease state, and (4) expectation of duration of life. Response categories from a prior report^45^ were modified for broader relevance to patients with both early and late-stage disease (See Supplementary Table 1). We summed responses to each item (coded 1 or 0 to reflect the presence or absence of understanding), yielding a score ranging from 0 to 4. Participants with perfect scores reflecting complete illness understanding (4 of 4 correct responses) were compared to all others.

*Symptoms of anxiety* were measured using the Generalized Anxiety Disorder 2-item questionnaire (GAD-2), a validated instrument that asks about symptoms of anxiety using a two-week recall period from 0 (“not at all”) to 3 (“nearly every day”), with higher scores indicating more anxiety^46,47^. Following established cut-points^46,48^, we characterized participants with scores of 3 or more as having symptoms of anxiety.

*Satisfaction with cancer care* was measured using the FAMCARE short-form, a validated 10-item instrument that assesses emotional support, personalization of care, support of decision-making, accessibility, and coordination^49,50^. Response categories of “very satisfied” (2 points), “satisfied” (1 point), or “not satisfied” (0 points) are summed to yield a total score ranging from 0 to 20, with higher scores reflecting greater satisfaction.

### Participant characteristics

Patient and care partner socio-demographic factors, health status, and employment were assessed in baseline telephone interviews. Health literacy was assessed using a validated single-item screening question^51^. Patient quality of life was assessed using the FACT-B^52^. The patient disease stage was assessed and adjudicated from information in the electronic health record at baseline and follow-up.

### Analysis

A sample size of 120 dyads was selected for this initial study envisioning a one-year enrollment to assess feasibility and acceptability and generate efficacy estimates in preparation for larger definitive trials. Descriptive statistics were used to examine differences between potentially eligible patients and those who enrolled in the study, and between intervention and control participants. The pre-specified time point for outcomes was 9 months. Participants were asked to reconsent to complete a final 12-month assessment, which is presented herein for completeness. As attrition for reasons other than death at 9 months was trivial (≤1.5%), we do not compare participants by the completion of 9-month assessments and focus on a complete case analysis of dyads in which both patients and care partners completed assessments at baseline and 9 months follow-up.

Engagement of care partners in electronic interactions was measured by patient portal (MyChart) registration and use. Symptoms of anxiety and complete illness understanding at 9 months were compared to baseline using logistic regression models estimated with generalized estimating equations (GEE) and an exchangeable correlation specification. Each model included group assignment, time (9 months versus baseline), and their interaction. Satisfaction with cancer care at 9 months was compared to baseline using linear regression models with the difference in the score as the outcome and a term for group assignment as the main independent variable. We additionally conducted a subanalysis among intervention dyads to comparatively examine outcomes by active care partner engagement as measured by whether care partners logged into the patient portal at least one time between enrollment and 9-months, referred to as “actively engaged,” with those who did not log into the patient portal. All models also included terms for patient education, care partner gender, and patient disease stage at baseline when comparing intervention to control, and patient disease stage and care partner employment status when comparing active to non-active intervention. Statistical analyses were performed with SAS statistical software, version 9.4 (SAS, Cary, NC) and all reported *p*-values are two-sided. *P* < 0.05 was considered statistically significant and no adjustments for multiple comparisons were performed.

### Reporting summary

Further information on research design is available in the Nature Research Reporting Summary linked to this article.

## Supplementary information

Supplementary Information

Reporting Summary Checklist

## Data Availability

The datasets that support the findings of this study are kept in institutional file storage on an internal server at the Johns Hopkins Bloomberg School of Public Health, and therefore are not publicly available in order to protect patient privacy. The data will be made available on reasonable request from the corresponding author for up to and including 5 years from the publication of the related manuscript, as described in the following metadata record: 10.6084/m9.figshare.13281254^53^. For all data requests please contact Dr Jennifer Wolff, Department of Health Policy and Management, Johns Hopkins Bloomberg School of Public Health, Baltimore, MD. jwolff2@jhu.edu.

## Supplementary information

**Supplementary information** The online version contains supplementary material available at 10.1038/s41523-021-00217-9.
