# Supplementary material for: A randomized intervention involving family to improve communication in breast cancer care
Source: NPJ Breast Cancer. 2021 Feb 12;7:14. doi: 10.1038/s41523-021-00217-9 (PMC7881185; doi:10.1038/s41523-021-00217-9)
Supplement: Supplementary file 1 — Supplementary Information [file 41523_2021_217_MOESM1_ESM.pdf]

Supplementary Figure 1. CONSORT Checklist

CONSORT 2010 checklist of information to include when reporting a randomised trial*

Section/Topic	Item No	Checklist item	Reported on page No
**Title and abstract**			
	1a	Identification as a randomised trial in the title	1
	1b	Structured summary of trial design, methods, results, and conclusions (for specific guidance see CONSORT for abstracts)	2
**Introduction**			
Background and objectives	2a	Scientific background and explanation of rationale	3
	2b	Specific objectives or hypotheses	3
**Methods**			
Trial design	3a	Description of trial design (such as parallel, factorial) including allocation ratio	9-10
	3b	Important changes to methods after trial commencement (such as eligibility criteria), with reasons	no changes
Participants	4a	Eligibility criteria for participants	10
	4b	Settings and locations where the data were collected	9-10
Interventions	5	The interventions for each group with sufficient details to allow replication, including how and when they were actually administered	10-12
Outcomes	6a	Completely defined pre-specified primary and secondary outcome measures, including how and when they were assessed	3 & 10
	6b	Any changes to trial outcomes after the trial commenced, with reasons	no changes
Sample size	7a	How sample size was determined	10
	7b	When applicable, explanation of any interim analyses and stopping guidelines	not applicable
Randomisation:			
Sequence generation	8a	Method used to generate the random allocation sequence	9
	8b	Type of randomisation; details of any restriction (such as blocking and block size)	9
Allocation concealment mechanism	9	Mechanism used to implement the random allocation sequence (such as sequentially numbered containers), describing any steps taken to conceal the sequence until interventions were assigned	9
Implementation	10	Who generated the random allocation sequence, who enrolled participants, and who assigned participants to interventions	9
Blinding	11a	If done, who was blinded after assignment to interventions (for example, participants, care providers, those	not applicable

		assessing outcomes) and how	
	11b	If relevant, description of the similarity of interventions	not relevant
Statistical methods	12a	Statistical methods used to compare groups for primary and secondary outcomes	10-11
	12b	Methods for additional analyses, such as subgroup analyses and adjusted analyses	not applicable
**Results**			
Participant flow (a diagram is strongly recommended)	13a	For each group, the numbers of participants who were randomly assigned, received intended treatment, and were analysed for the primary outcome	3-4
	13b	For each group, losses and exclusions after randomisation, together with reasons	3-4
Recruitment	14a	Dates defining the periods of recruitment and follow-up	8-9
	14b	Why the trial ended or was stopped	8
Baseline data	15	A table showing baseline demographic and clinical characteristics for each group	Table 1 (page 1[illegible]
Numbers analysed	16	For each group, number of participants (denominator) included in each analysis and whether the analysis was by original assigned groups	Figure 1 (page 1[illegible]
Outcomes and estimation	17a	For each primary and secondary outcome, results for each group, and the estimated effect size and its precision (such as 95% confidence interval)	3-5
	17b	For binary outcomes, presentation of both absolute and relative effect sizes is recommended	3-5
Ancillary analyses	18	Results of any other analyses performed, including subgroup analyses and adjusted analyses, distinguishing pre-specified from exploratory	3-5
Harms	19	All important harms or unintended effects in each group (for specific guidance see CONSORT for harms)	4
**Discussion**			
Limitations	20	Trial limitations, addressing sources of potential bias, imprecision, and, if relevant, multiplicity of analyses	8
Generalisability	21	Generalisability (external validity, applicability) of the trial findings	8
Interpretation	22	Interpretation consistent with results, balancing benefits and harms, and considering other relevant evidence	7
**Other information**			
Registration	23	Registration number and name of trial registry	9
Protocol	24	Where the full trial protocol can be accessed, if available	12
Funding	25	Sources of funding and other support (such as supply of drugs), role of funders	13

*We strongly recommend reading this statement in conjunction with the CONSORT 2010 Explanation and Elaboration for important clarifications on all the items. If relevant, we also recommend reading CONSORT extensions for cluster randomised trials, non-inferiority and equivalence trials, non-pharmacological treatments, herbal interventions, and pragmatic trials. Additional extensions are forthcoming: for those and for up to date references relevant to this checklist, see www.consort-statement.org.

Supplementary Figure 2. Patient-Family Agenda Setting Checklist

Making the most of your visit

This conversation guide is for patients who attend medical oncology visits with a family member or friend. Together, use this guide to prepare for today's visit.

STEP 1 **How can your family member or friend be most helpful today?**

Together, decide what types of help you would like. Mark (✓) all that apply:

- ☐ Listen and remember what the doctor says or means.
- ☐ Prompt you to ask questions or tell the doctor your concerns.
- ☐ Ask the doctor questions or give the doctor information directly.
- ☐ Allow you time alone with the doctor for some or all of the visit.

STEP 2 **What do you want to discuss with your doctor today?**

Together, decide which concerns are most important.

Mark (✓) if a concern to:

Patient Health Issues	PATIENT	FAMILY
Treatment goals and expectations	☐	☐
Who and how to ask questions about medical concerns, appointments, or tests	☐	☐
Symptoms or side effects (pain, nausea, fatigue)	☐	☐
Nutrition (weight gain or loss) and lifestyle	☐	☐
Stress, worry, feeling sad or blue	☐	☐
Sexuality, intimacy, reproductive issues	☐	☐
Coming to terms with breast cancer	☐	☐
Concerns about school, work, finances	☐	☐
Chances of cancer recurrence or spread	☐	☐
Planning for potential worsening of disease	☐	☐
Add other issues: (optional)	☐	☐
	☐	☐

STEP 3 **How do you want to manage the patient's health information after today's visit?** *If you are interested, the study staff will show you how to use MyChart after today's visit.*

Mark (✓) all that apply:

- ☐ Set up access to MyChart (electronic access to patient's health record).
- ☐ Set up family/friend "shared" (proxy) access to patient's MyChart account.
- ☐ I am not interested in MyChart.

Note. Figure was previously published in *Breast Cancer Research and Treatment*: Breast Cancer Res Treat. 2019 Aug; 177(1): 127–136. Published online 2019 Jun 4. doi: 10.1007/s10549-019-05306-9

Supplementary Table 1. Illness Understanding Response Categorization

Illness Understanding Question	Response Categories	Early	Metastatic
1) How would you describe your current **health status**? I am	1 Healthy and have no symptoms<br>2 Have symptoms but likely to improve<br>3 Have symptoms and likely to get worse<br>4 Have symptoms and likely to remain stable<br>5 Don't Know	1 Correct<br>2 Correct<br>3 Incorrect<br>4 Correct<br>5 Incorrect	1 Incorrect<br>2 Incorrect<br>3 Correct<br>4 Correct<br>5 Incorrect
2) Which of the following best represents what your medical oncology health care team have told you about a **cure** for your cancer?	1 My cancer is expected to be cured<br>2 My cancer may be cured if treatments are successful<br>3 My cancer cannot be cured but may be controlled for a while with treatment<br>4 My cancer cannot be cured and is no longer responding to treatment<br>5 I Don't Know	1 Correct<br>2 Correct<br>3 Incorrect<br>4 Incorrect<br>5 Incorrect	1 Incorrect<br>2 Incorrect<br>3 Correct<br>4 Correct<br>5 Incorrect
3) What **stage** is your cancer?	2 Early stage cancer (Stage 1-2)<br>3 Locally advanced stage cancer (Stage 3)<br>4 Advanced stage cancer (Stage 4/Metastatic)<br>5 Don't Know	2 Correct<br>3 Correct<br>4 Incorrect<br>5 Incorrect	2 Incorrect<br>3 Incorrect<br>4 Correct<br>5 Incorrect
4) When you think about your **life**, do you think in terms of:	1 Months<br>2 Years<br>3 Decades<br>4 Don't Know	1 Incorrect<br>2 Correct<br>3 Correct<br>4 Incorrect	1 Correct<br>2 Correct<br>3 Incorrect<br>4 Incorrect
