# Supplementary material for: A randomized intervention involving family to improve communication in breast cancer care
Source: NPJ Breast Cancer. 2021 Feb 12;7:14. doi: 10.1038/s41523-021-00217-9 (PMC7881185; doi:10.1038/s41523-021-00217-9)
Supplement: Supplementary file 2 — Reporting Summary Checklist [file 41523_2021_217_MOESM2_ESM.pdf]

nature research

Corresponding author(s): Jennifer L. Wolff, PhD
Antonio C. Wolff, MD FACP, FASCO

Reporting Summary

Nature Research wishes to improve the reproducibility of the work that we publish. This form provides structure for consistency and transparency in reporting. For further information on Nature Research policies, see Authors & Referees and the Editorial Policy Checklist.

Statistical parameters

When statistical analyses are reported, confirm that the following items are present in the relevant location (e.g. figure legend, table legend, main text, or Methods section).

n/a	Confirmed	
☐	☒	The exact sample size (*n*) for each experimental group/condition, given as a discrete number and unit of measurement
☐	☒	An indication of whether measurements were taken from distinct samples or whether the same sample was measured repeatedly
☐	☒	The statistical test(s) used AND whether they are one- or two-sided<br>*Only common tests should be described solely by name; describe more complex techniques in the Methods section.*
☐	☒	A description of all covariates tested
☐	☒	A description of any assumptions or corrections, such as tests of normality and adjustment for multiple comparisons
☐	☒	A full description of the statistics including central tendency (e.g. means) or other basic estimates (e.g. regression coefficient) AND variation (e.g. standard deviation) or associated estimates of uncertainty (e.g. confidence intervals)
☐	☒	For null hypothesis testing, the test statistic (e.g. *F*, *t*, *r*) with confidence intervals, effect sizes, degrees of freedom and *P* value noted<br>*Give P values as exact values whenever suitable.*
☒	☐	For Bayesian analysis, information on the choice of priors and Markov chain Monte Carlo settings
☒	☐	For hierarchical and complex designs, identification of the appropriate level for tests and full reporting of outcomes
☒	☐	Estimates of effect sizes (e.g. Cohen's *d*, Pearson's *r*), indicating how they were calculated
☒	☐	Clearly defined error bars<br>*State explicitly what error bars represent (e.g. SD, SE, CI)*

Our web collection on statistics for biologists may be useful.

Software and code

Policy information about availability of computer code

Data collection	Data collected from telephone interviews were captured by research staff using REDCap, version 10.0.10. Patient and care partners' utilization of MyChart was assessed using date-and time-stamped audit trails of patient portal actions extracted by the Clinical Research Data Acquisition Core of the Johns Hopkins Institute for Clinical and Translational Research.
Data analysis	Statistical analyses were performed with SAS statistical software, version 9.4, (Cary, NC).

For manuscripts utilizing custom algorithms or software that are central to the research but not yet described in published literature, software must be made available to editors/reviewers upon request. We strongly encourage code deposition in a community repository (e.g. GitHub). See the Nature Research guidelines for submitting code & software for further information.

Data

Policy information about availability of data

All manuscripts must include a data availability statement. This statement should provide the following information, where applicable:

- Accession codes, unique identifiers, or web links for publicly available datasets
- A list of figures that have associated raw data
- A description of any restrictions on data availability

The data supporting the related manuscript are kept in institutional file storage on an internal server at the Johns Hopkins Bloomberg School of Public Health. There

are de-identification concerns in small, regionally restricted, clinical datasets that prevent these data being openly available, but data will be made available at reasonable request from the corresponding author for up to and including 5 years from publication of the related manuscript.
For all data requests please contact:
Dr. Jennifer Wolff, Department of Health Policy and Management, Johns Hopkins Bloomberg School of Public Health, Baltimore, MD. jwolff2@jhu.edu

Field-specific reporting

Please select the best fit for your research. If you are not sure, read the appropriate sections before making your selection.

☐ Life sciences ☒ Behavioural & social sciences ☐ Ecological, evolutionary & environmental sciences

For a reference copy of the document with all sections, see nature.com/authors/policies/ReportingSummary-flat.pdf

Behavioural & social sciences study design

All studies must disclose on these points even when the disclosure is negative.

Study description	We examined effects of a communication intervention to engage family care partners on patient portal (MyChart) use, illness understanding, satisfaction with cancer care, and symptoms of anxiety. We conducted a single-blind randomized trial of patients in treatment for breast cancer. All outcomes were quantitative. Patient and care partner outcomes were assessed independently.
Research sample	The study sample includes patients with early and advanced breast cancer from two separate clinics (urban and suburban) within a single academic institution with a spectrum of patients that is representative of community practice. There were 118 dyads with complete assessments at 9-months. Enrolled patients had a mean age of 53.5 years. Most were women (99.2%) with high school education or more (87.3%). About half (56.8%) were diagnosed with early stage disease.
Sampling strategy	Recruitment letters were mailed to patients of participating clinicians and screening calls were fielded to determine eligibility. A sample size of 120 dyads was selected for this initial study envisioning a one year enrollment to assess feasibility and acceptability and generate efficacy estimates in preparation for larger definitive trials. Our intent was indeed to enroll up to 132 participants (along with their care partners) expecting approximately 120 pairs (dyads) would be evaluable for the primary outcomes. Our final analytic dataset includes 118 complete pairs (dyads), which closely aligns with our original target sample.
Data collection	Patient portal use for both patients and care partners was assessed from date and time-stamped interactions reflecting the frequency, timing, and type of MyChart interactions. Illness understanding was measured by 4 questions regarding knowledge that is considered to be essential to making informed treatment decisions in serious illness. Symptoms of anxiety were measured using the Generalized Anxiety Disorder 2-item questionnaire (GAD-2). Satisfaction with cancer care was measured using the FAMCARE short-form, a validated 10-item instrument that assesses emotional support, personalization of care, support of decision-making, accessibility, and coordination. Trained research staff fielded a standardized telephone survey to patients and care partners one week post-enrollment and at 3, 9, and 12 months. All telephone surveys were audio-recorded and conduced privately between the interviewer and the participant. The interviewer was not blind to the experimental group due to intervention-specific questions included in the survey.
Timing	Patient and care partner dyad enrollment began in August 2017 and was completed in November 2018. Follow-up interviews continued through November 2019.
Data exclusions	As attrition for reasons other than death at 9 months was trivial (≤1.5%), we focus on a complete case analysis of dyads in which both patients and care partners completed assessments at baseline and 9 months follow-up. Among the n=132 patient-family dyads enrolled in the study, there were n=118 dyads with complete assessments at 9-months. Data from n=14 dyads were excluded from the analyses due to incomplete assessments that arose from patient death (n=10), withdrawn consent (n=3), or loss to follow up (n=1).
Non-participation	Recruitment letters were mailed to 361 patients of participating clinicians. Twenty (5.5%) returned a mailed card indicating that they were not eligible (n=11; 3.0%) or declining participation (n=9; 2.5%); 21 (5.8%) could not be reached. Screening calls were fielded to 320 patients of whom 139 (43.4%) were not eligible and 49 (15.3%) refused participation: 132 (41.3%) patient-companion dyads were eligible and agreed to participate. Among the n=132 patient and care partner dyads enrolled in the study, n=3 (2.3%) withdrew consent before the 9-month assessment. Participants who withdrew consent cited not being interested in completing surveys for time commitment or other personal reasons.
Randomization	Dyads were randomized using stratified, blocked randomization with alternating block sizes of 4 and 6 for each clinician. Dyads assigned to the intervention were provided a paper version of our patient-family agenda setting checklist to complete together at the point of care, immediately in advance of a regularly scheduled medical oncology visit. Upon completion of the checklist, front desk staff were instructed to implement participant's stated patient portal registration preferences. After the visit, intervention patients and care partners were provided paper handouts with instructions on how to access MyChart and clinical visit notes and offered facilitated registration by research staff in the clinic. Dyads assigned to the control group received usual care. Usual care refers to availability of MyChart registration that may be self-initiated by patients and care partners under standard clinic protocol.

Reporting for specific materials, systems and methods

Materials & experimental systems

n/a	Involved in the study	
☒	☐	Unique biological materials
☒	☐	Antibodies
☒	☐	Eukaryotic cell lines
☒	☐	Palaeontology
☒	☐	Animals and other organisms
☐	☒	Human research participants

Methods

n/a	Involved in the study	
☒	☐	ChIP-seq
☒	☐	Flow cytometry
☒	☐	MRI-based neuroimaging

Human research participants

Policy information about studies involving human research participants

Population characteristics	All models included terms for patient education, care partner gender, and patient disease stage at baseline when comparing intervention to control, and patient disease stage and care partner employment status when comparing active to non-active intervention.
Recruitment	Oncology clinicians at participating clinics provided informed consent indicating their permission for the study team to contact their patients. Patients of participating clinicians who were in active treatment for early stage or advanced breast cancer were mailed letters describing the study three weeks before their next scheduled visit. Patients who did not "opt out" by mail were contacted by research staff to discuss study procedures and administer a screening interview. Patients undergoing active breast cancer therapy were eligible if they reported regularly attending appointments with a family or unpaid care partner who also agreed to participate. Enrolled patients were younger than those who did not participate (53.9 versus 56.8 years; p=0.045) with no other differences observed.
